# Nodule sous cutané révélant un angioléiomyome

**DOI:** 10.11604/pamj.2013.15.48.2823

**Published:** 2013-06-08

**Authors:** Kbira El Morabite, Baderddine Hassam

**Affiliations:** 1 Service de Dermatologie, CHU Ibn Sina, Université Med V, Souissi, Rabat, Maroc

**Keywords:** Angioléiomyome, tumeur bénigne, nodule sous cutané, Angioleiomyoma, benign tumor, subcutaneous nodule

## Image en médecine

L'angioléiomyome est une tumeur bénigne musculaire lisse rare comportant une composante vasculaire. L'angioléiomyome est encore connu sous le nom de léiomyome vasculaire ou d'angiomyome. Il se présente en général sous la forme d'une lésion sous-cutanée solitaire et douloureuse, localisée au niveau des extrémités, le plus souvent aux membres inférieurs, en particulier au niveau du mollet et à la cheville. Le pic d'incidence se situe entre la quatrième et la sixième décennie, avec une prédominance féminine. Le diagnostic de l'angioléiomyome est histologique. L’évolution d'un léiomyome vasculaire est classiquement bénigne. Sans transformation maligne ni récidive après une exérèse chirurgicale complète. Nous rapportons le cas d'une patiente âgée de 26 ans, sans antécédents pathologiques particuliers qui présente depuis 1 an un nodule sous cutané au niveau du mollet gauche. La consultation était motivée par le caractère gênant du nodule et la douleur au contact. A l'examen clinique, elle présentait un nodule sous cutané de 2 cm de grand axe, sensible avec une peau en regard d'aspect normal. La patiente a bénéficié d'une exérèse complète du nodule. L’étude histologique a révélée la présence d'une formation nodulaire bien circonscrite faite de fibres musculaires lisses agencées en bandes compactes, celles-ci sont sillonnées par de nombreuses sections vasculaires à paroi fine. Cette prolifération est délimitée en périphérie par des faisceaux musculaire lisses et montre au niveau de la zone centrale une dégénérescence hyaline et myxoide. Le diagnostic d'angioléiomyome était retenu.

**Figure 1:**
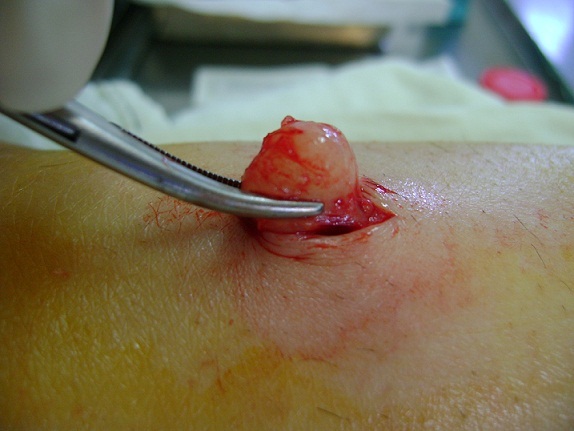
Formation tumorale blanchâtre sous cutanée de 1cm de diamètre au niveau du mollet gauche

